# Supplementary material for: ChatGPT as a Tool for Biostatisticians: A Tutorial on Applications, Opportunities, and Limitations
Source: Stat Med. 2025 Oct 23;44(23-24):e70263. doi: 10.1002/sim.70263 (PMC12548020; doi:10.1002/sim.70263)
Supplement: Supplementary file 1 — Data S1. Supporting Information. [file SIM-44-0-s001.zip › Supplementary Materials Additional.zip!/3.7_Translation_Between_Multiple_Statistical_Programming_Languages/3.7.1_R_to_python/3.7.1_Translation_Chat5.pdf]

Run 5

Clear memory

I don't actually have memory on in this chat, so I'm not storing any information you've shared. If you'd like to manage or clear memory in the future, you can do that by going to **Settings > Personalization > Memory**.

Want help finding it or have questions about how memory works?

Take the role of an expert biostatistician for this chat

Absolutely — I'll step into the role of an expert biostatistician for this chat. Feel free to throw any questions my way, whether they're about study design, statistical modeling, data analysis, clinical trials, survival analysis, or anything else in the biostats realm. What are we working on today?

Translate the following code into python:

```
library(MASS)
set.seed(1)
temp <- tempfile()
download.file(
  "https://archive.ics.uci.edu/static/public/17/breast+cancer+wisconsin+diagnostic.zip",
  temp
)
data <- read.table(unz(temp, "wdbc.data"), sep = ",")
data$V2 <- data$V2 == "M"
unlink(temp)

train_index <- sample(seq_len(nrow(data)), round(nrow(data) * 0.8))

train <- data[train_index, -1]
test <- data[-train_index, -1]

base_model <- glm(V2 ~ 1, data = train, family = binomial)


step_model <- stepAIC(
  base_model,
  direction = "both",
  scope = list(
```

```
    lower = V2 ~ 1,
    upper = V2 ~ V3 + V4 + V5 + V6 + V7 + V8 + V9 + V10 + V11 +
      V12 + V13 + V14 + V15 + V16 + V17 + V18 + V19 + V20 + V21 +
      V22 + V23 + V24 + V25 + V26 + V27 + V28 + V29 + V30 + V31
  ),
  # scope = formula(V2 ~ .),
)

summary(step_model)
test_pred <- predict(step_model, test, type = "response")

sum((test_pred > 0.5) == test$V2) / nrow (test)
names(coef(step_model))
```

Here's the full Python equivalent of your R code using **pandas**, **scikit-learn**, and **statsmodels**, closely mirroring the functionality of `stepAIC` from the **MASS** package (via forward-backward stepwise logistic regression):

```python
import pandas as pd
import numpy as np
import statsmodels.api as sm
from sklearn.model_selection import train_test_split
from urllib.request import urlretrieve
import zipfile
import tempfile
import os

# Set seed
np.random.seed(1)

# Download and read the data
temp = tempfile.NamedTemporaryFile(delete=False)
urlretrieve(
    "https://archive.ics.uci.edu/static/public/17/breast+cancer+wisconsin+diagnostic.zip",
    temp.name
)

with zipfile.ZipFile(temp.name, 'r') as zip_ref:
    zip_ref.extractall(tempfile.gettempdir())

data = pd.read_csv(os.path.join(tempfile.gettempdir(), "wdbc.data"), header=None)
data[1] = data[1] == "M"  # Convert to boolean: True for malignant

# Train-test split (80-20)
train, test = train_test_split(data, test_size=0.2, random_state=1)

# Remove ID column
train = train.drop(columns=[0])
test = test.drop(columns=[0])

# Base model: intercept only
y_train = train[1].astype(int)
X_train = train.drop(columns=[1])
X_test = test.drop(columns=[1])
y_test = test[1].astype(int)

# Stepwise logistic regression function
def stepwise_selection(X, y,
                       initial_list=[],
                       threshold_in=0.01,
                       threshold_out=0.05,
                       verbose=True):
    included = list(initial_list)
```

```
    while True:
        changed = False
        # forward step
        excluded = list(set(X.columns) - set(included))
        new_pval = pd.Series(index=excluded, dtype=float)
        for new_column in excluded:
            model = sm.Logit(y, sm.add_constant(pd.DataFrame(X[included + [new_column]]))).fit(disp=
            new_pval[new_column] = model.pvalues[new_column]
        best_pval = new_pval.min() if not new_pval.empty else None
        if best_pval is not None and best_pval < threshold_in:
            best_feature = new_pval.idxmin()
            included.append(best_feature)
            changed = True
            if verbose:
                print(f'Add  {best_feature:<30} with p-value {best_pval:.6f}')

        # backward step
        if included:
            model = sm.Logit(y, sm.add_constant(pd.DataFrame(X[included]))).fit(disp=0)
            pvalues = model.pvalues.iloc[1:]  # exclude intercept
            worst_pval = pvalues.max()
            if worst_pval > threshold_out:
                worst_feature = pvalues.idxmax()
                included.remove(worst_feature)
                changed = True
                if verbose:
                    print(f'Drop {worst_feature:<30} with p-value {worst_pval:.6f}')
        if not changed:
            break
    return included

# Run stepwise selection
selected_features = stepwise_selection(X_train, y_train, verbose=False)

# Final model
X_train_selected = sm.add_constant(X_train[selected_features])
model = sm.Logit(y_train, X_train_selected).fit()
print(model.summary())

# Prediction
X_test_selected = sm.add_constant(X_test[selected_features])
pred_probs = model.predict(X_test_selected)
pred_labels = (pred_probs > 0.5).astype(int)
accuracy = (pred_labels == y_test).mean()
print(f"\nTest Accuracy: {accuracy:.4f}")

# Print selected feature names
print("\nSelected features in final model:")
print(model.params.index.tolist())
```

Key Notes:

- `` `stepAIC` `` in R performs stepwise selection based on AIC. In Python, we approximate it with p-value based forward-backward selection using `` `statsmodels` ``.
- The `` `Logit` `` model used here corresponds to `` `glm(..., family = binomial)` `` in R.
- We manually replicate the stepwise procedure since Python does not natively include an exact `` `stepAIC` `` equivalent.

Let me know if you want a version that uses **AIC** explicitly instead of p-values, or want to visualize the coefficients, ROC curve, or confusion matrix.

Use AIC instead

Great — to replicate **R's `stepAIC` using AIC explicitly** in Python, we'll modify the stepwise procedure to add/drop features based on **model AIC**, just like `MASS::stepAIC`. Here's the revised Python implementation:

```python
import pandas as pd
import numpy as np
import statsmodels.api as sm
from sklearn.model_selection import train_test_split
from urllib.request import urlretrieve
import zipfile
import tempfile
import os

# Set seed
np.random.seed(1)

# Download and extract data
temp = tempfile.NamedTemporaryFile(delete=False)
urlretrieve(
    "https://archive.ics.uci.edu/static/public/17/breast+cancer+wisconsin+diagnostic.zip",
    temp.name
)

with zipfile.ZipFile(temp.name, 'r') as zip_ref:
    zip_ref.extractall(tempfile.gettempdir())

data = pd.read_csv(os.path.join(tempfile.gettempdir(), "wdbc.data"), header=None)
data[1] = data[1] == "M"  # Convert to boolean (malignant)

# Train-test split (80-20)
train, test = train_test_split(data, test_size=0.2, random_state=1)
train = train.drop(columns=[0])
test = test.drop(columns=[0])

# Define predictors and response
y_train = train[1].astype(int)
X_train = train.drop(columns=[1])
X_test = test.drop(columns=[1])
y_test = test[1].astype(int)

# AIC-based stepwise selection
def stepwise_aic(X, y, verbose=True):
    included = []
    best_aic = np.inf
    while True:
        changed = False
        excluded = list(set(X.columns) - set(included))
        candidates = []

        # Try adding features
        for col in excluded:
            model = sm.Logit(y, sm.add_constant(X[included + [col]])).fit(disp=0)
            candidates.append((model.aic, included + [col], f"add {col}"))

        # Try removing features
        for col in included:
            subset = [c for c in included if c != col]
            if subset:
                model = sm.Logit(y, sm.add_constant(X[subset])).fit(disp=0)
                candidates.append((model.aic, subset, f"drop {col}"))

        if not candidates:
            break

        # Pick the model with lowest AIC
        aic_values = [c[0] for c in candidates]
        min_idx = np.argmin(aic_values)
        best_candidate_aic, best_candidate_vars, action = candidates[min_idx]

        if best_candidate_aic + 1e-6 < best_aic:  # small tolerance for float comparison
```

```
            if verbose:
                print(f"{action:<12} -> AIC: {best_candidate_aic:.2f}")
            included = best_candidate_vars
            best_aic = best_candidate_aic
            changed = True

        if not changed:
            break

    return included

# Run stepwise AIC selection
selected_vars = stepwise_aic(X_train, y_train, verbose=True)

# Final model
X_train_final = sm.add_constant(X_train[selected_vars])
final_model = sm.Logit(y_train, X_train_final).fit()
print(final_model.summary())

# Evaluate on test data
X_test_final = sm.add_constant(X_test[selected_vars])
pred_probs = final_model.predict(X_test_final)
pred_labels = (pred_probs > 0.5).astype(int)
accuracy = (pred_labels == y_test).mean()

print(f"\nTest Accuracy: {accuracy:.4f}")
print("\nFinal model features:")
print(final_model.params.index.tolist())
```

What this does:

- Iteratively adds or removes variables based on **AIC minimization**, matching what `` `stepAIC` `` in R does.
- Uses a small tolerance (`` `1e-6` ``) to avoid float precision issues in AIC comparisons.
- Stops when no single-step change reduces the AIC.

Let me know if you want a visual (like an AIC trace plot), ROC curve, or this turned into a reusable function/module.
